# Early Exposure to Environmental Pollutants: Imidacloprid Potentiates Cadmium Toxicity on Zebrafish Retinal Cells Death

**DOI:** 10.3390/ani12243484

**Published:** 2022-12-09

**Authors:** Davide Di Paola, Enrico Gugliandolo, Fabiano Capparucci, Marika Cordaro, Carmelo Iaria, Rosalba Siracusa, Ramona D’Amico, Roberta Fusco, Daniela Impellizzeri, Salvatore Cuzzocrea, Rosanna Di Paola, Rosalia Crupi, Alessio Filippo Peritore

**Affiliations:** 1 Department of Chemical, Biological, Pharmaceutical and Environmental Science, University of Messina, 98166 Messina, Italy; 2 Department of Veterinary Science, University of Messina, 98166 Messina, Italy; 3 Department of Biomedical and Dental Sciences and Morphofunctional Imaging, University of Messina, 98166 Messina, Italy; 4 Department of Pharmacological and Physiological Science, School of Medicine, Saint Louis University, Saint Louis, MO 63103, USA

**Keywords:** environment contaminant, apoptosis, pesticides

## Abstract

**Simple Summary:**

Simultaneous exposure to several contaminants can create a cross-linkage that leads to increased fish toxicity. Cadmium (Cd), one of the most common metals detected in the environment, can cause concentration-dependent damage in developing fish. Co-exposure of Cd and other contaminants, such as the pesticide imidacloprid (IMI), can cause developmental damage in zebrafish, particularly to eye cells. In fact, co-exposure to concentrations that are in themselves non-toxic can cause morphological damage accompanied by a sharp increase in apoptosis and cell death in retinal cells, with disruption of the antioxidant mechanism.

**Abstract:**

In the present study, we analyzed the combination of non-toxic concentrations per se, of Cd and a pesticide the imidacloprid (IMI) (10 and 50 μM for Cd and 195 μM for IMI), to highlight early developmental toxicity and possible damage to retinal cells. Co-exposure to Cd and IMI showed a toxic effect in zebrafish larval development, with lowered degrees of survival and hatching, and in some cases the induction of structural alterations and edema. In addition, co-exposure to 50 and 195 μM, respectively, for Cd and IMI, also showed increased apoptosis in eye cells, accompanied by up regulation of genes associated with antioxidant markers (cat, sod1, nrf2 and ho-1). Thus, the present study aims to highlight how the presence of multiple contaminants, even at low concentrations, can be a risk factor in a model of zebrafish (*Danio rerio*). The presence of other contaminants, such as IMI, can cause an enhancement of the toxic action of Cd on morphological changes in the early life stage of zebrafish, but more importantly disrupt the normal development of the retina, eventually triggering apoptosis.

## 1. Introduction

Among the various families of pesticides, neonicotinoids are the most widely used because of their low molecular weight to high water solubility. Despite the European restrictions of 2013, they are produced in large quantities and used in agriculture in various ways [1,2,3]. Within the family of neonicotinoids, Imidacloprid (IMI) is one of the most used and detected at environmental level as a result of agricultural use in concentrations of several hundred mg/L, while in water bodies the percentages were lower about ng/L [4]. IMI residues in water can be harmful to aquatic organisms. Low concentrations of IMI about 1 μg/L, are capable of having shown effects on aquatic insects, but not on other species, such as mollusks, algae, fish, or amphibians. Insects are the most vulnerable organism group, followed by crustaceans, while fish appear several orders of magnitude less sensitive to direct short-term exposure of IMI [5,6]. Several studies in models such as zebrafish and medaka have shown the toxicity of IMI in a stage-dependent manner. Reduced locomotion has been reported in zebrafish larvae continuously exposed to IMI from fertilization for up to five days [7]. Exposure to IMI on medaka resulted in reduced growth of adults and juveniles after long-term exposure in mesocosms [5]. Recently, it has been shown that the simultaneous exposure of IMI along with other environmental contaminants can cause toxicity in the early developmental stages of zebrafish [8].

Aquatic organisms in natural environments are commonly exposed to chemical mixtures rather than individual compounds [9,10]. Increases in agricultural and industrial activities in an area directly influences the quality of water. In other words, water reservoirs are collectors of all materials spread by human industrial and agricultural activities.

The high use of metals such as cadmium, mercury, and lead, in industry and consumer products has led to an increase in their presence in the environment. Having contaminated water, heavy metals (HMs) accumulate in organisms, which are consumed by fish or penetrate fish directly through skin and gill later [5,6]. HMs cause the mutation of fish inner organs, disturb immune reactions, change blood parameters, reduce an organism’s adaptation qualities, vitality, resistance to diseases. Furthermore, several organs can be targets of HMs toxicity, such as the retina, an important component of the central nervous system (CNS). Indeed, lead and cadmium have shown depressive effects on the response to the rod potential [11]. Prolonged exposure to heavy metals can induce oxidative stress resulting in increased production of reactive oxygen species (ROS). The increased production of ROS, which have different targets (such as proteins, lipids, or DNA), can lead to various diseases as well as cell death and aging. Previous studies have shown that exposure to Cd in conjunction with other contaminants at nontoxic concentrations can cause increased oxidative stress in fish [12,13]. Therefore, the aim of this work was to evaluate the toxic effects of the concomitant presence of the pollutants, which are often present numerous despite the small concentrations reported.

## 2. Materials and Methods

### 2.1. Zebrafish Maintenance and Embryo Collection

Wild type (WT) mature zebrafish with an age of 6 months have been used to produce embryos. The University of Messina Center of Experimental Fish Pathology (Center for Experimental Ichthyopathology of Sicily, CISS, Messina, Italy) supplied zebrafish maintenance and fertilized egg collection. The fish were fed dry and live food twice a day at a rate of 3% of their body weight (BW). Mature females and males were mated in a 2:1 ratio for successful reproduction. The eggs were collected the next day in a chamber at 28 °C, bleached, and non-fertilized eggs were discarded. For the experiments, only embryos that had reached the blastula stage were employed. The FET (Fish Embryo Toxicity) test was carried out in accordance with OECD guidelines [14] and ISO 15088.

### 2.2. Survival Rate, Hatching Rate and Morphology Score

Healthy embryos were implanted in 24-well culture plates at 4 h post fertilization (hpf) (1 embryo in 2 mL solution/well). Cadmium chloride (CdCl2. 2H2O) and Imidacloprid (PESTANAL^®^) were purchased from Sigma (St. Louis, MO, USA) and prepared on reconstituted water. Cd and IMI was dissolved in distilled and deionized water to produce 10 mM stock solutions. Before application, both stock solutions were diluted in fresh embryo embryo medium to generate the final concentrations needed, Cd 10 and 50 μM and IMI 195 μM (Embryo medium was composed with 15mM NaCl, 0.5 mM KCl, 1 mM CaCl2, 1 mM MgSO4, 0.15 mM KH2PO4, 0.05 mM Na2HPO4, 0.7 mM NaHCO3, pH 7.3).

Zebrafish embryos were exposed to different concentration of Cd and IMI alone and in combination for 24–120 hpf to measure the toxic effects over a continuing observation period. Fertilized eggs were transferred into 24-well plates with test solutions and incubated at 28 °C at a 14:10 h day/night light regime. The median lethal concentration (LC50), median combined adverse effect concentration (EC50) and relevant cadmium exposure doses were previously determined [15]. The LC50 and EC50 were 168 μM and 138 μM, respectively. Briefly, embryos were exposed to water only (blank control); Cadmium at nominal concentrations of 10 and 50 μM, IMI 195 μM alone and combinate (20 replicates for each experimental group; three independent experiments). Cd and IMI solutions were daily changed and the entire survival rate and developmental abnormalities of embryos and larvae were monitored and photo-recorded at 24, 48, 72 and 96 hpf [16]. During the period of exposure to the substances the embryos/larvae were observed every 24 h for abnormalities in development, survival, and hatching rate, as well as morphological changes [17]. Morphology scores were determined at 96 hpf as previously described. Nine endpoints, including body shape, somites, notochord, tail, fins, heart, face, brain, and pharyngeal arches/jaws, were examined to evaluate the phenotypes of the zebrafish, and eight larval specimens per group were used for scoring [18].

### 2.3. Gene Expression Analysis

The total RNA from 20 homogenized zebrafish larvae for each gropu was isolated in 0.50 mL TRIzol reagent (Invitrogen, Waltham, MA, USA) according to the manufacturer’s instructions. Total RNAwas isolated according to the manufacturer’s instructions. The ratio of absorbance at 260–280 nm, as well as the banding patterns on a 1% agarose formaldehyde gel, were used to verify the quality ofthe RNA in each sample. RNA quality was evaluated by gel electrophoresis, with the concentration measured with NanoDrop 2000 (Thermo Scientific Waltham, MA, USA iScript RT-PCR kit (Bio-Rad, Hercules, CA, USA) was used to synthesize first-strand cDNA according to manufacturer’s recommendations. The reverse transcription master mix was prepared adding to 1 μg of RNA template the iScript RT Supermix (5× RT supermix with RNase H+ Moloney (gray cap, 25 or 100 reactions) murine leukemia virus (MMLV) reverse transcriptase, RNase inhibitor, dNTPs, oligo(dT), random primers, buffer, MgCl2 and stabilizers) and the nuclease-free water. Briefly, 1 μg/μL total RNA was mixed with 5 μL of 5× iScript^®^ Reaction Mix and 1 μL of iScript^®^ Reverse Transcriptase and RNase-free water to make the final volume of 25 μL. The complete reaction mix was incubated in a thermal cycler (Priming 5 min at 25 °C, Reverse transcription 20 min at 46 °C, RT inactivation for one minute at 95 °C). Real-time PCR was performed with a 20-μl volume containing 10 μL of 1× SsoFast EvaGreen Supermix (Bio-Rad, Hercules, CA, USA), 1 μL of cDNA, 7 μL of RNase/DNase-free water, and 500 nM each primer. PCR conditions were: initial denaturation at 95 °C for 15 min, followed by 45 cycles of amplification at 95 °C for 20 s and 60 °C for 40 s. Final extension at 60 °C for 60 s and ahold at 4 °C were then performed on StepOnePlus Real-Time PCR System (Applied Biosystems, Waltham, MA, USA). All the qPCR reactions were performed with three parallel samples; the negative control contains no sample template. Table 1 shows the detailed information on the primers as previously reported [19,20,21]. β-actin was used as an internal control for normalizing relative expression levels between samples [22,23,24]. Data analysis was performed using the 2^−∆∆Ct^ method and the results are expressed as fold changes.

### 2.4. Acridine Orange and Terminal Deoxynucleotidyl Transferase dUTP Nick End Labeling (TUNEL)

The cell death was measured based on the reported methods modified by Zou et al. [25]. Embryos were transferred into 24-well plates at 120 hpf and treated with acridine orange for 30 min (10 μg/mL). Zebrafish larvae were rinsed in fresh embryo medium five times and anesthetized before visualization. TUNEL staining protocol was conducted in agreement with the manufacturer, Roche, as previously described [21]. The larval sections included in the paraffin were deparaffinized in xylene and were rehydrated by a series of alcohols at decreasing percentages of ethanol, permeabilized with 0.1 M citrate buffer, and then incubated in TUNEL reaction mixture for 60 min at 37 °C in the dark. The tissue was rinsed in PBS several times and was observed using exciting wavelengths under stereomicroscope (Leica M0205C, multifocal).

### 2.5. Morphological Assessment

Larvae were observed under a Leica M0205C, multifocal (*n* = 20 per treatment) for measurements of eye diameter at 120 hpf. The diameter of the whole eye was defined as the distance from the pigmented epithelium of one pole to the opposite pole paralleled with the spine. For the lens, the diameter was defined as the distance from the lens epithelium of one pole to the opposite pole paralleled with the spine. All images were analyzed using ImageJ (version 1.47).

### 2.6. Detection of Reactive Oxygen Species (ROS) and Lipid Peroxidation

ROS detection and malondialdehyde (MDA) analysis were performed as previously described [26]. Briefly, at 96-h postfertilization (hpf), zebrafish larvae were homogenized in 800 μL CytoBuster protein extraction buffer (Novagen, San Diego, CA, USA) using an Ultra-Turrax T8 basic homogenizer (IKA, Staufen, Germany). The homogenates were centrifuged at 12,000× *g* for 10 min in 4 °C, and the supernatants were collected for various assays. ROS concentrations were assessed using the oxidant-sensitive probe 2,7-dichlorofluorescein diacetate (DCF-DA; Sigma-Aldrich, St Louis, MO, USA). The fluorescence intensity was measured using a microplate reader (Molecular Device, M2, Union City, CA, USA) with excitation and emission at 485 and 530 nm, respectively. The ROS concentration was expressed in arbitrary units (DCF/mg protein). Lipid peroxidation was detected as malondialdehyde (MDA) reacting with thiobarbituric acid to form a colored complex by spectrofluorometric analysis. The level of MDA is expressed as nanomoles per milligram protein.

### 2.7. Statistical Evaluation

All values in the figures and text are expressed as the mean ± standard error of the mean (SEM) of N number of experiments. The results were analyzed by two-way for graphs where there were two variables such as time and different exposures (e.g., survival rate), or one way ANOVA followed by a Tukey post-hoc test for multiple comparisons. The data were tested for normal distribution with the Shapiro-Wilk test (*p* < 0.05) and they were represented as mean ± standard error of the mean (SEM), (alpha value of 0.05). Statistical analysis was performed using the Graphpad Prism 8.

## 3. Results

### 3.1. Survival Rate, Hatching Rate and Morphology

The toxic effects of Cd on zebrafish embryonic development, alone and in combination with IMI, have been evaluated at different concentrations based on previous studies were applied to observe morphology, survival, and hatching rate of embryos/larvae (Table 2. Exposure to Cd at 10 and 50 μM showed no toxic effects on survival and morphological changes in embryos and larvae from 24 up to 96 hpf, while the high concentration, 50 and 195 μM, showed a significant decrease compared with the control group. Besides decreasing the survival rate, the combination at the concentrations of 50 and 195 μM caused several morphological deficits, such as scoliosis and yolk edema. Moreover, even the two single exposure of Cd 10 and 50 μM, than IMI exposure at 195 μM, did not show toxic effects on embryos and larvae. The combination of Cd and IMI, but only at 50 μM + 195 μM showed a significant decrease of survival on embryos and larvae from 48 to 96 hpf, while Cd 10 μM + IMI 195 μM group did not show effect on lethal endpoints. Furthermore, the effect of Cd and IMI exposure on hatching rate, another parameter usually used for toxicity studies in zebrafish, was evaluated. We observed a delay in hatching only in the group in combination of Cd and IMI at the concentrations of 50 and 195 μM, at 72 and 96 hpf. The exposures of individual Cd and IMI concentrations showed no effect whatsoever on the hatching rate.

### 3.2. Cell Apoptosis

**Figure 1:**
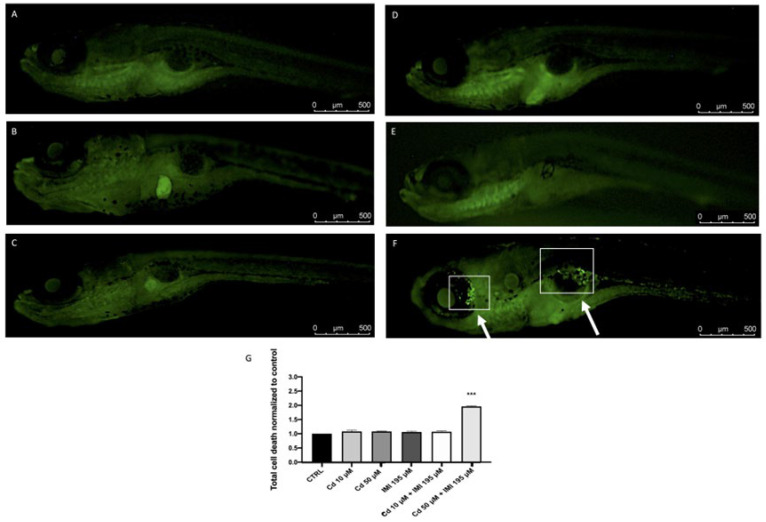
The effects of Cd and IMI on total ell death in zebrafish embryos. (**A**) Control; (**B**) Cd 10 μM; (**C**) Cd 50 μM; (**D**) IMI 195 μM; (**E**) Cd 10 μM + IMI 195 μM; (**F**) Cd 50 μM + IMI 195 μM; Cell death death (**G**). At 120 hpf, Cd 50 μM, IMI 195 μM and Cd 50 μM + IMI 195 μM incubation, the levels of total cell death were observed and photographed by a fluorescence microscope after staining with acridine orange. Values are expressed as means ± SEM of three independent experiment data; *** at *p* < 0.001 against CTRL.

AO staining showed that the difference in apoptotic signals between control and co-treated groups were significant for the high concentrations group at 120 hpf. The apoptotic signals increased in a dose-dependent manner for the combination exposure. Compared to less apoptotic signal in zebrafish eyes and yolk from the control group, a significant increase in apoptosis was observed in co-treated group with high concentration. In particular, the eyes showed an important increase of cell death after co-treated of Cd 50 μM and IMI 195 μM, while the single exposure did not show this significant increase compared to control group (Figure 1).

**Figure 2:**
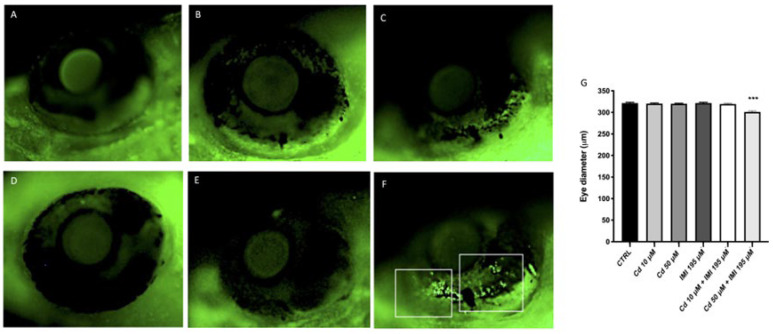
Detection of Cd and IMI exposure indued changes in the cell death within zebrafish eyes. Cell apoptosis within the eyes of live zebrafish embryos at 120 hpf determined using acridine orange (AO) staining. (**A**) Control; (**B**) Cd 10 μM; (**C**) Cd 50 μM; (**D**) IMI 195 μM; (**E**) Cd 10 μM + IMI 195 μM; (**F**) Cd 50 μM + IMI 195 μM; Cell death signals were indicated by green fluorescent spot on a indicated by the blank square (F); (**G**) Effect Cd and IMI on eye diameter at 120 hpf. Values are expressed as means ± SEM of three independent experiment data; *** at *p* < 0.001 against CTRL.

Moreover, we observed a reduction in the eye diameters on Cd and IMI co-exposed larvae followed in a dose-dependent relationship by 120 hpf (Figure 2). Indeed, the measurement of eye size showed a reduction in size only in the group with co-exposure of Cd and IMI to the highest concentrations 50 and 195 μM; while single exposures of Cd and IMI, as well as co-exposure at low concentrations 10 and 195 μM, showed no significant reductions in diameter.

**Figure 3:**
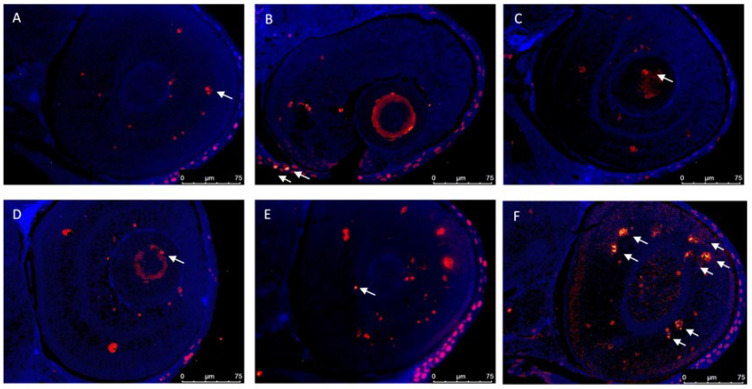
TUNEL assays indicated an abnormal apoptotic pattern. TUNEL-positive apoptotic cells (white arrow) in zebrafish embryos treated with Cd and IMI at 120 hpf. (**A**) Control; (**B**) Cd 10 μM; (**C**) Cd 50 μM; (**D**) IMI 195 μM; (**E**) Cd 10 μM + IMI 195 μM; (**F**) Cd 50 μM + IMI 195 μM.

The TUNEL fluorescence assay is a well-established, fast, and simple nonradioactive technique to detect and quantify neurons undergoing apoptosis. It detects free 3′-OH termini in single-stranded breaks in high-molecular-weight nuclear DNA fragments. The presence of these groups is considered an established marker of apoptosis. Apoptosis is a physiological mechanism of cell renewal, thus present under homeostatic conditions, as shown in Figure 3, in the control group. Similar results were observed using the TUNEL assay. At 120 hpf, the combination of Cd and IMI resulted in significant differences in TUNEL labeling within zebrafish eyes compared to control group. TUNEL labeling increased significantly after co-treatment of Cd 50 μM and IMI 195 μM, but not for the single exposure.

### 3.3. Gene Expression

**Figure 4:**
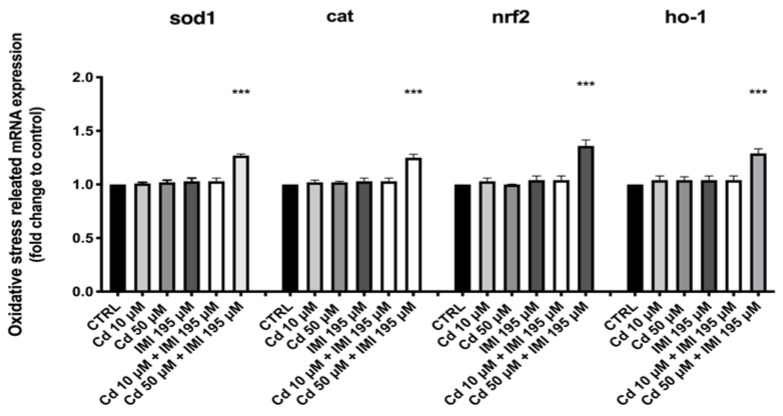
Effects of Cd and IMI exposure on the mRNA levels of stress oxidative pathway sod1, cat, nrf2 and ho-1 in larval zebrafish. Values are expressed as means ± SEM of three independent experiment data; *** at *p* < 0.001 against CTRL.

To further compare the effects of Cd and IMI on the antioxidant system, gene expression related to the antioxidant system was examined, and the results are shown in Figure 4. Cd and IMI co-exposure induced an up-regulation of sod1 and cat genes. Both heavy metal single exposures, as well as single IMI exposure, did not increase levels of sod1 and cat mRNA at 120 hpf compared with control groups. The multifunctional regulator nuclear factor erythroid 2-related factor (Nrf2) regulates the expression of genes that code for proteins involved in numerous pathways, including antioxidant pathways. In addition, Cd and IMI combined exerted influences on Nrf2 gene expression, but not for the single exposure. In fact, the co-exposure with Cd and IMI showed an up-regulation of Nrf2 mRNA levels. Moreover, one of the genes regulated through Nrf2 is heme oxygenase-1(ho-1). As observed for nrf2 gene, ho-1 gene also showed an up-regulation following co-exposure of Cd and IMI, an increase that is not found in single exposures of the two compounds compared with the control group.

### 3.4. Lipid Peroxidation and Reactive Oxygen Species(ROS) Formation

**Figure 5:**
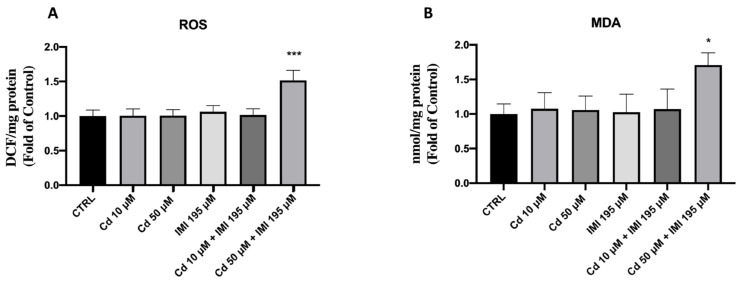
Effects of Cd and IMI exposure in ROS formation (**A**) and MDA (**B**) in zebrafish. Values are expressed as means ± SEM of three independent experiment data; * at *p* < 0.05 against CTRL *** at *p* < 0.001 against CTRL.

Larvae exposed to Cd and IMI combination at 50 μM and 195 μM resulted in a significant increase of ROS production in at 96 hpf compared to control (Figure 5A), while the single exposure with Cd and IMI, as well as the combination of Cd and IMI at 50 μM and 195 μM, did not show any significant changes in the PFOS exposure groups (Figure 5B). Moreover, we assessed the lipid peroxidation through Malondialdehyde (MDA) levels. Figure 5 illustrates the MDA content in the larval zebrafish increased significantly after the exposure to Cd 50 μM and IMI 195 μM mixture at 96 h, compared to the control group. While there was no effect on MDA content increased after single Cd and IMI, as well as in the group of Cd 10 μM and IMI 195 μM mixture (Figure 5).

## 4. Discussion

Previous studies have demonstrated the adverse effects of IMI on aquatic organisms; in fact, lethal and sublethal effects have been reported on several aquatic species with developmental body structural alterations [27,28]. Fish are the primary target in the aquatic environment of human-caused contaminants and pollutants, such as pesticides [29,30]. Pesticides, which pollute natural waters through agricultural runoff and various other means, can cause fish to die or decrease their numbers causing an ecological imbalance [31,32,33]. Zebrafish embryos and larvae have been widely used in the study of in vivo responses following the exposure to environmental contaminant, as well as HMs or drug [21,34,35,36,37,38]. Various quantitative studies have established the potential of zebrafish as a model for HM-induced cell death during embryogenesis [39]. Several studies have shown that the co-presence of different pesticides and contaminants can result in increased toxicity in zebrafish larvae [9,40]. Therefore, we should consider the toxic effects of individual pesticides and contaminants as well as their mixtures in the risk management of pesticides. In zebrafish, mortality and malformations are the most important indices used to assess the toxicological effects of environmental agents. Both Cd and neonicotinoid IMI induced increased mortality and malformations in a dose-dependent manner [8,12,41]. The current study demonstrated that co-exposure of Cd and IMI in the early life stages of zebrafish could increase the toxicity of the individual contaminants. The concentrations of Cd showed no mortality of larvae at 120 hpf, confirming past studies. Hatching is a critical time in zebrafish embryogenesis; therefore, the decrease in hatching rate was induced by functional and structural disturbances during embryonic development [42,43]. In addition, the suppression of embryogenesis, or the inhibition of mitosis, or the inability of embryonic larvae to open the chorion possibly contributed to the developmental delay [44,45]. Several studies have shown how Cd, even at low bumps, can cause renal dysfunction, carcinogenicity, and cardiovascular disease, in animal studies. During the gastrulation period, exposure to Cd can be critical, and if prolonged even through the segmentation period can result in the higher incidence of malformations. In the present study, it has also been seen that Cd and IMI in combination may exhibit dose-dependent toxicity in zebrafish embryonic development; we observed at 96 hpf an alterations teratogenic effect by increasing the incidence of malformations, such as scoliosis and yolk edema.

Environmental pollutants are well-known inducers of ROS, result in an imbalance between ROS production, and scavenging by endogenous antioxidants can directly or indirectly disturb physiological functions of many cellular macromolecules such as DNA, protein, and lipids, and activate cellular stress-sensitive signaling pathways Moreover, another important mechanism involved in the pollutants toxicity is lipid peroxidation. MDA is the primary by-product of lipid peroxidation (LPO), and an increase in its content embodies the degree of cellular damage caused by free radicals [46]. Our data demonstrate that Cd and IMI co-exposure is capable of inducing ROS production, and at the same time increase MDA content during early zebrafish development. Furthermore, it is well known that the production of ROS in fish in response to contamination is intimately associated with apoptotic cell death. Embryonic development may be especially sensitive to ROS-induced oxidative stress [47]. Apoptosis is a highly regulated cell death process. Ectopic apoptotic cells can be found in malformed tissues and mutants. Genetic mutations or toxicant exposures lead to developmental abnormalities, resulting in the disruption of apoptosis [48]. Previous studies showed that zebrafish mutant embryos with neurodegeneration formed an abnormal central nervous system and have ectopic apoptosis in their neural tissues [49]. Cadmium induces apoptosis or necrosis in rat cortical neuron cultures in a dose-dependent manner [50]. A high number of apoptotic cells throughout the body is reported in the anterior and posterior ends of zebrafish embryos having axial defect after cadmium treatment [51]. In the present study, Cd and IMI co-exposed embryos develop small eyes, a malformation not observed in the single exposures. Furthermore, during early eye development prior to differentiation of the committed lens primordial cells, and the initiation of retina neuronal production, a significant cell death in the eyes of Cd and IMI co-exposed embryos was observed.

Eyes have been reported to be sensitive to cadmium in humans and animals [52]. For example, Cd present in cigarettes is a major cause of cataracts and age-related macular degeneration, all of which lead to blindness [53]. Cd appears to cause retinal damage on zebrafish embryos and larvae at concentrations lower than the EC50 and LC50 [15,54] In fact, Cd exposure results in increased blindness-related symptoms, a loss of sun neurons, retinal ganglion neurons, and cone photoreceptors. However, these toxic effects of Cd appear to be concentration-dependent when analyzed individually [54]. Although the concentrations analyzed in the present study are higher than those found in the environment, the comorbidity of Cd and other potentially toxic substances in the environment can be a huge risk factor [55,56,57,58]. In the present study, we have shown how there is an increased toxicity of Cd when present together with other environmental contaminants. The toxic action of Cd is enhanced by the co-presence of IMIs, as opposed to single exposures of these two compounds. Here, we can only postulate that cell death may be one of the causes of retinogenesis reduction, and hence the formation of small eyes. How cadmium induces ectopic apoptosis in the eye, remain unclear. However, the possibility to find Cd as a contaminant along with other substances increases the focus on potential toxic effects even for concentrations that are themselves without effect. Because fish have a poor metabolic rate and clearance of heavy metals, the effects of Cd may be persistent in zebrafish [59].

Critical pathways are involved in the developmental toxicity, such as oxidative stress and inflammation [60,61]. A mixture of contaminant in aquatic environments leads to the induction of several metabolic processes and the increase in the production of ROS in aquatic organisms [62,63]. In antioxidant defense mechanisms, cat and sod1 have vital functions as the first barriers against oxidative stress triggered by ROS generation [62]. However, when the ROS-scavenging system cannot effectively neutralize or eliminate the excess of ROS, oxidative damage occurs [64]. In the present study, the mRNA expression levels of the antioxidant genes, cat and sod1, were significantly increased by exposure to Cd and IMI at single nontoxic concentration. On the contrary, these indicators were not significantly affected by Cd and IMI alone, which may explain with a non-sufficient toxic concentration did not induce oxidative damage. Nrf2 dissociate into the nucleus and bind to antioxidant response elements of antioxidant genes to up-regulate their mRNA expression [65]. In addition, Nrf2 regulates the cellular antioxidant response by binding ARE sequence in the promoter region of antioxidant enzyme genes and activating gene expression [66]. We found that Nrf2 and HO-1 were up-regulated after Cd and IMI exposure. More interestingly, only the combination at 50 and 195 μM showed an up-regulation of Nrf2 and HO-1, suggesting a dose-dependent activity of cd in the synergistic activation of oxidative pathway.

## 5. Conclusions

Our data have shown how sublethal concentrations of Cd and IMI when co-exposed can cause damage in the early stages of zebrafish development. Cd exposure, which is associated with developmental damage to retinal cells, is exacerbated when co-exposed to sublethal doses of IMI, inducing increased apoptosis and an imbalance in normal oxidative stress regulatory mechanisms. Since little scientific evidence exists to date on the potential cross-linkage between these two contaminants, further research will be needed to understand the mechanisms of their interactions and the risks of their presence in water.

## Figures and Tables

**Table 1:** Primers for Real-Time PCR.

Gene	Primer Orientation	Nucleotide Sequence
*β-actin*	forward	5′- CTTCCAGCAGATGTGGATCA -3′
	reverse	5′- GCCATTTAAGGTGGCAACA-3′
*sod1*	forward	5′- GGCCAACCGATAGTGTTAGA -3′
	reverse	5′- CCAGCGTTGCCAGTTTTTAG -3′
*cat*	forward	5′- AGGGCAACTGGGATCTTACA -3′
	reverse	5′- TTTATGGGACCAGACCTTGG -3′
*nrf2*	forward	5-AAGCAGACGGAGGAGGAG -3
	reverse	5′- GGAGGTGTTCAGGCAAGG-3′
*ho-1*	forward	5′- AAGCAAAGCGGCAGAGAAC-3′
	reverse	5′- TGGAGCAGTCAGATGAAGTGT-3′

**Table 2:** Cd and IMI exposure effects.

	Survival				Hatching				Morphology
	24 h	48 h	72 h	96 h	24 h	48 h	72 h	96 h	96 h
**CTRL**	100 ± 0	98.00 ± 1	97.67 ± 1.20	97.67 ± 1.20	0	22.67 ± 1.45	100 ± 0	100 ± 0	ND
**Cd 10 μM**	100 ± 0	98.00 ± 1	97.67 ± 1.20	97.67 ± 1.20	0	22.67 ± 1.45	99.67 ± 0.33	99.67 ± 0.33	ND
**Cd 50 μM**	100 ± 0	99.67 ± 0.33	99.67 ± 0.33	99.67 ± 0.33	0	22 ± 1.15	99.67 ± 0.33	99.67 ± 0.33	ND
**IMI 195 μM**	100 ± 0	99.67 ± 0.33	99.67 ± 0.33	99.67 ± 0.33	0	22.33 ± 1.76	99.67 ± 0.33	99.67 ± 0.33	ND
**Cd 10 μM +** **IMI 195 μM**	100 ± 0	97.33 ± 0.88	97.00 ± 1	97.00 ± 1	0	22.67 ± 1.45	99.67 ± 0.33	99.67± 0.33	ND
**Cd 50 μM +** **IMI 195 μM**	100 ± 0	95.00 ± 0.57 **	88.33 ± 1.45 ***	83.33 ± 0.88 ***	0	22.33 ± 1.45	80.67 ± 1.52 ***	93.00 ± 1.73 ***	SC, YE

Values are expressed as percentage of embryos/larvae examined in the study. SC = Scoliosis, YE = Yolk sac Edema, ND = Not Detected. Values are expressed as means ± SEM of three independent experiment data; ** at *p* < 0.01 against CTRL. *** at *p* < 0.001 against CTRL

## Data Availability

All data included in this study are available upon request by contacting the corresponding author.
